# Association of *PPM1G* methylation with risk-taking in alcohol use disorder

**DOI:** 10.1038/s41598-020-62504-y

**Published:** 2020-03-26

**Authors:** Chun Il Park, Hae Won Kim, Syung Shick Hwang, Jee In Kang, Se Joo Kim

**Affiliations:** 1 Institute of Behavioral Science in Medicine, Yonsei University College of Medicine, Seoul, Republic of Korea; 2 Department of Psychiatry, Yonsei University College of Medicine, Seoul, Republic of Korea; 3 Department of Medical Education, Yonsei University College of Medicine, Seoul, Republic of Korea; 4 Graduate School, Yonsei University College of Medicine, Seoul, Republic of Korea

**Keywords:** Epigenetics, Diagnostic markers

## Abstract

Alcohol use disorder (AUD) is a chronic and relapsing disease with a substantial genetic influence. Given the recent discovery of the association of *PPM1G* methylation with alcohol use disorder (AUD) from a genome-wide methylation study, we sought to verify and extend the previous work of AUD-related impulsivity in a Korean population with AUD. A total of 244 men with AUD were assessed for psychological characteristics and behavioral impulsivity using stop signal task (response inhibition) and Balloon Analog Risk Task (risk-taking). Leukocyte DNA methylation at *PPM1G* was quantified using pyrosequencing. The effects of *PPM1G* methylation on severity of problematic drinking measured by Alcohol Use Disorder Identification Test (AUDIT) and multidimensional impulsivity were tested using linear regression analyses. Hypermethylation of *PPM1G* was significantly associated with risk-taking propensity among men with AUD. No significant association of *PPM1G* methylation was found to be associated with AUDIT scores and response inhibition. Our findings indicate that altered methylation of *PPM1G* may influence the impulsive choice of risk-taking in AUD. Further research is required in order to determine the role of *PPM1G* in the pathophysiology of AUD and multidimensional impulsivity.

## Introduction

Alcohol use disorder (AUD) is a chronic relapsing disease characterized by excessive alcohol drinking and loss of control over alcohol consumption. Approximately 50% of the risk of AUD can be attributed to genetic factors^1^. Based on the results from genome-wide association studies of AUD, it is suggested that hundreds of variants with small genetic effects across the genome contribute to the genetic susceptibility of AUD^2^. In addition to a genetic predisposition, growing evidence supports the theory that environmental factors, such as early life stress, increases vulnerability for the development and psychopathological conditions of AUD^3,4^. Childhood adversity has been reported to be associated with early initiation of alcohol^5^ and higher suicidal attempts in patients with AUD^6^. These findings suggest that the interplay between genes and environment is involved in the pathophysiology of AUD.

Epigenetic modifications such as DNA methylation (reversible modulation of gene expression without changing the DNA sequence), can be key to understanding these gene-environment interactions. A genome-wide DNA methylation study with 18 monozygotic twin pairs discordant for AUD discovered a differentially methylated region in the 3’-protein-phosphatase-1G (*PPM1G*) gene that is considered to be potentially involved in modulating cortical transmission to the striatum through dephosphorylation of the metabotropic glutamate receptors^7^. That study also showed that the *PPM1G* DNA methylation level was associated with *PPM1G* gene expression, and *PPM1G* hypermethylation was correlated with an escalation in daily alcohol drinking as well as an impulsivity trait, measured by self-reported impulsivity and brain activity during behavioral inhibition task, in independent adolescent samples^7^.

Given the recent discovery of an epigenetic association of the *PPM1G* gene with AUD, we sought to verify and extend the previous work of AUD-related impulsivity in a Korean clinical population with AUD. Since the impulsivity trait, an endophenotype for AUD, is a multifaceted construct^8,9^, we assessed multidimensional impulsivity using a self-report measure and behavioral tasks, including impulsive action of response inhibition and impulsive choice of risk-taking, and examined their associations with the *PPM1G*. To ascertain an unbiased effect of the *PPM1G*, we controlled for the effects of potential confounders such as childhood adversity on epigenetic modulation.

## Methods

### Participants

A total of 244 male subjects with AUD were included in this study. All participants were recruited from inpatients psychiatric facilities for detoxification and rehabilitation of AUD and assessed after detoxification treatment. Diagnosis of AUD was confirmed by psychiatrists using a structured clinical interview for the Diagnostic and Statistical Manual of Mental Disorders, 4th Edition (DSM-IV-TR) criteria and clinical observation. Most recent drinking confirmed by clinician was at least 7 days before study enrollment, and no signs of alcohol intoxication or withdrawal symptoms were observed in any participant on the study day of self-report questionnaire and tasks. Exclusion criteria were: (1) being admitted primarily for another major psychiatric disorder; (2) having an intellectual disability; and (3) having other substance dependencies except tobacco use. In addition, subjects with mood disorders including major depressive disorder based on the DSM-IV-TR criteria and clinician’s careful observation were excluded. Participants were ethnically all Korean. Ethnicity was determined based on self-reported ethnicity and the administrator’s observations. All patients who participated in this study provided written informed consent according to the procedures approved by the Severance Hospital Institutional Review Board (Seoul, Korea), and all protocols were performed in accordance with approved guidelines.

### Measurements

The severity of problematic drinking in patients with AUD was assessed using the Alcohol Use Disorders Identification Test (AUDIT)^10^. Higher AUDIT scores indicated a more harmful drinking behavior. The Korean version of the Barratt Impulsiveness Scale (BIS) was also used to assess participants’ self-reported impulsivity. To evaluate childhood trauma, we used the modified Korean version of the Parent-Child Conflict Tactics Scale (mPCCTS)^11^ which is based on the Parent-Child Conflict Tactics Scale^12^. The severity of depressive and anxiety symptoms was assessed using the Beck Depression Inventory (BDI)^13^ and the Beck Anxiety Inventory (BAI)^14^. All questionnaires had been validated in the Korean population previously^11,15–18^.

### Behavioral tasks for impulsive action and impulsive choice

The stop signal task (SST) was used to assess the impulsive action of response inhibition^19^. The stop-signal reaction time (SSRT) was estimated. A higher SSRT value indicates a worse inhibitory control. In addition, the Balloon Analog Risk Task (BART)^20^, a computerized decision-making test, was conducted to assess the impulsive choice of risk-taking. Risk-taking propensity was measured by calculating the mean number of pumps in trials during which the balloons did not explode, a higher BART score represents a greater risk-taking propensity.

### Pyrosequencing of PPM1G

Venous blood samples were collected in 4 ml EDTA vacutainers and stored at −80 °C prior to analysis. Genomic DNA was isolated from whole blood using the standard techniques. Three cytosine-guanine (CpG) sites in the 3’ untranslated region (UTR) of *PPM1G* were selected and designed using PSQ Assay Design software (Qiagen^TM^) based on the previous study^7^. The genomic locations of the three selected CpG sites were as follows: CpG1 (Chr2: 27,604,246; GRCh37), CpG2 (Chr2: 27,604,258; GRCh37), and CpG3 (Chr2: 27,604,271; GRCh37). Pyrosequencing was used for DNA methylation analysis by *Genomictree, Inc*. The DNA was bisulfite treated and sequencing was performed using the Pyro Gold reagents kit (Qiagen, Hilden, Germany).

### Statistical analyses

The SPSS 25.0 software for Windows (SPSS Inc., Chicago, IL, USA) was used for analyses of all data in this study. Descriptive statistical analyses were performed for various variables including demographic, clinical, and epigenetic characteristics of the participants in this study. Multivariable linear regression analyses with the ‘enter method’ were used to test the associations of the *PPM1G* methylation level with the AUDIT scores and multidimensional impulsivity (self-reported impulsivity; BIS score, impulsive action; SST, and impulsive choice; BART) after adjusting for multiple potential confounders. The regression model included the *PPM1G* methylation level, childhood trauma (high vs. low), and other demographic and clinical variables (age, duration of AUD, depressive symptoms, and anxiety levels), that may influence the relationship between the *PPM1G* and AUD-related phenotypes, as independent variables. Childhood trauma exposure was categorized as a binary value, high and low, using a median split of mPCCTS scores. The mean value of methylation levels at the three CpG sites in *PPM1G* was used for analyses.

## Results

### Demographic and clinical characteristics

Socio-demographic, clinical characteristics and the methylation level of the *PPM1G* of the study participants are summarized in Table 1. All participants were men with AUD, and further detailed descriptions are presented in Table 1.

**Table 1:** Demographic, clinical, psychological characteristics and *PPM1G* methylation status of patients with AUD.

Variable	^a^AUD (n = 244)
Age, years	45.87 ± 6.68
Duration of AUD, years	16.03 ± 9.33
**Clinical characteristics**	
AUDIT	27.13 ± 7.55
BDI	18.09 ± 12.41
BAI	13.50 ± 11.01
mPCCTS	2.89 ± 3.30
**Multidimensional impulsivity trait**	
BIS	51.03 ± 10.49
^b^SSRT	173.38 ± 136.53
BART	28.53 ± 15.36
***PPM1G*** **methylation (%)**	
CpG1	89.54 ± 3.06 ^c^(82.17, 99.92)
CpG2	96.44 ± 3.03 ^c^(85.28, 100)
CpG3	94.48 ± 1.03 ^c^(87.05, 97.99)

AUD, alcohol use disorder; AUDIT, the Alcohol Use Disorders Identification Test; BDI, Beck.

Depression Inventory; BAI, Beck Anxiety Inventory; BIS, Barratt Impulsiveness Scale;

mPCCTS, modified Parent-Child Conflict Tactics Scale; SSRT, Stop Signal Reaction Time.

BART, Balloon Analog Risk Task.

^a^Mean ± standard deviation;

^b^N = 221;

^c^(Minimum, Maximum).

### Regression model predicting the severity of problematic drinking and multidimensional impulsivity

A regression model demonstrated no significant association of the *PPM1G* with AUDIT scores. Among the potential associated factors selected as independent variables by the regression model, young age, long duration of AUD, higher BDI scores and presence of childhood adversity were significantly associated with higher AUDIT scores (Table S1).

For multidimensional impulsivity, a regression model of impulsive action showed a significant association between *PPM1G* and risk-taking propensity as measured by the BART (Table 2). In this model, *PPM1G* methylation explained 3.5% of the variance in risk-taking propensity, indicating small to moderate effect size. Hypermethylation of the *PPM1G* was correlated with higher risk-taking propensity. On the other hand, a regression model of the BIS score showed no significant association of the *PPM1G* with self-reported impulsivity trait. In that model, only depressive symptom severity was significantly associated with self-reported impulsivity (Table S2). In addition, a regression model of impulsive choice showed no significant association of the *PPM1G* with response inhibition by SSRT value (Table S3).

**Table 2:** Multiple linear regression analysis^a^ with enter method for predicting the risk-taking propensity (BART) in patients with AUD (N = 244).

	B	SE	β	T	P
Constant	−142.712	63.758		−2.238	0.026
Age	−0.296	0.162	−0.129	−1.821	0.07
Duration of AUD	−0.07	0.117	−0.043	−0.599	0.55
BDI	−0.005	0.1	−0.004	−0.046	0.963
BAI	−0.028	0.112	−0.02	−0.255	0.799
^b^Early life trauma	−0.315	2.04	−0.01	−0.154	0.877
^c^*PPM1G* methylation (%)	1.999	0.677	0.187	2.954	0.003

^a^Model summary: R^2^ = 0.059, adjusted R^2^ = 0.035, F_(6, 237)_ = 2.464, P = 0.025.

^b^High early life trauma was coded as 1, low early life trauma was coded as 0 based on the median value of mPCCTS.

^c^Mean value of methylation at three CpG sites in *PPM1G*.

BART, Balloon Analog Risk Task; AUD, alcohol use disorder; SE, Standard error; BDI, Beck Depression Inventory; BAI, Beck Anxiety Inventory.

## Discussion

The present study examined the role of *PPM1G* DNA methylation in multidimensional impulsivity among clinical samples with AUD, based on the novel finding of the *PPM1G* differentially methylated region from a genome-wide DNA methylation study of monozygotic twins discordant for AUD^7^. Our results demonstrated a significant association between *PPM1G* hypermethylation and the impulsive choice of risk-taking propensity after adjusting for the possible effects of variable confounders such as childhood adversity on epigenetic changes. When considering that the impulsive choice of risk-taking dramatically increases in early adolescence^21^ and is known to contribute to increase susceptibility for AUD^22^, our findings suggest that *PPM1G* hypermethylation may mediate the effect of high risk-taking in the pathophysiology of AUD.

Hypermethylation at the *PPM1G* region on chromosome 2 has been reported to be associated with lower mRNA levels of *PPM1G* in adolescent non-clinical samples^7^, although we did not examine the mRNA expression in this study. The previous finding indicates that *PPM1G* methylation levels influence *PPM1G* expression and protein levels. *PPM1G* is a member of the human protein phosphatase 2C (PP2C) family which has been implicated in cellular survival and stress response^23,24^. Studies in animals and *in vitro* work suggested a role of the *PPM1G* in regulating cell cycle progression and cellular stress response pathways^23^. Although little is known about the biological functioning of the *PPM1G* in humans, there is some indirect evidence suggesting a relationship between the *PPM1G* and AUD-related phenotypes. The region of chromosome 2p14‐2q14.3 has been linked to various psychiatric conditions including AUD and suicidal attempts, related to impulsivity and behavioral disinhibition^25–27^. A genetic study with polymorphisms across chromosome 2 in a case-control sample for alcoholism showed a significant association of rs2384629, a genetic polymorphism on the *PPM1G* gene, with combined AUD and suicide attempts or conduct phenotypes^25^. This suggests a genetic role of the *PPM1G* in AUD-related impulsivity. Ruggeri *et al*. reported a significant association of *PPM1G* hypermethylation with self-reported impulsivity measured by impulsivity-related subscales of the Substance Use Risk Profile Scale (SURP). This association was significant even after controlling for the possible genotype effects of polymorphisms covering the *PPM1G* locus of methylation status. Although the biological function of the *PPM1G* remained unclear, these findings suggest that the *PPM1G* may play a role in the pathophysiology of AUD, possibly through the impulsivity trait, an endophenotype for AUD.

Among our regression models predicting severity of problematic drinking or multidimensional impulsivity, the *PPM1G* methylation level was significant only for the impulsive choice of risk-taking, not for AUDIT scores, self-reported impulsivity, or impulsive action of behavioral inhibition using the stop signal task. Lack of significant association between *PPM1G* and AUDIT scores in the present study seemed to be consistent with the previous negative finding of alcohol exposure severity in non-clinical adolescents^7^. However, our sample consisted entirely of chronic alcohol-dependent patients with relatively high AUDIT scores. Therefore, the relationship between *PPM1G* methylation and severity of AUD needs to be confirmed in a cohort that includes the entire spectrum of AUDIT scores. For impulsivity, there was some discrepancy of self-reported impulsivity and behavioral inhibition between our study and Ruggeri’s results. However, direct comparison may be difficult due to a variety of differences including demographic factors such as age, sex, and ethnicity, clinical characteristics (e.g. clinical vs. non-clinical), measurements of impulsive action and choice, as well as the used self-report tools (e.g. BIS vs. SURP). Because impulsivity is a complex and multidimensional construct in nature^8,9^, comprehensive measure of multidimensional impulsivity would be beneficial to enhance the determination of the genetic effects on trait impulsivity and brain response related to impulsivity as an endophenotype for AUD.

Although biological mechanisms modulating methylation status of *PPM1G* is unknown, recent research suggests that excessive alcohol use can induce epigenetic dysregulation in human and rodent brains through alteration of the DNA methylation dynamics such as changes in DNA-methyltransferase (DNMT) expression and activity^28,29^. A postmortem brain study of bipolar disorder and schizophrenia suggested that chronic alcohol abuse has an effect on number of DNMT 1 mRNA-positive neurons^29^. Further research is needed to elucidate the biological mechanisms underlying regulation of DNA methylation of *PPM1G*.

There are several limitations to this study. First, we did not recruit healthy controls, as our main purpose was to examine epigenetic influence of *PPM1G* on quantitative AUD-related traits such as impulsivity among individuals with AUD. Future case control studies with healthy controls may help understand disease-specific methylation changes of *PPM1G* and their role in AUD. In addition, when considering sex differences in alcohol use and alcohol-related problems^30^, our findings in male patients may have limited application to female patients with AUD. Second, our study with a cross-sectional design restricts the ability to reveal a causal link of *PPM1G* methylation with the AUD-related impulsivity trait. Ruggeri *et al*.’s finding of a significant association of *PPM1G* hypermethylation with the escalation of daily alcohol drinking between ages 14 and 16 in non-clinical adolescents supports the possibility that *PPM1G* hypermethylation may be a vulnerable marker for developing AUD, rather than a consequence of AUD. Future longitudinal research is warranted in order to establish the direction of causality in their relationship. Third, we did not consider the effect of some potential confounders, including heterogeneity in white blood cell composition^31^ and cigarette smoking^32,33^, that may contribute to inter-individual variability in DNA methylation. In particular, since smoking can induce epigenetic modifications, the possible effect of smoking on DNA methylation should be considered in future studies. Fourth, since we cannot measure DNA methylation levels directly in the brain during clinical studies, we assumed that the DNA methylation pattern of *PPM1G* in peripheral leukocytes reflects the *PPM1G* methylation pattern in brain. Although DNA methylation is tissue-specific, there is some evidence suggesting that patterns of peripheral DNA methylation or mRNA expression reflect changes in brain tissues^34,35^. In addition, peripheral *PPM1G* methylation levels have been reported to be associated with brain activity during the impulsive action task of the SST^7^. However, it is still questionable whether peripheral DNA methylation could serve as surrogates for DNA methylation in the brain. Finally, the present sample size may be limited to detect an association between DNA methylation levels at a given CpG site and a complex phenotype controlled by many genes with very small effect. Therefore, a study with a larger number of samples with stronger statistical power is needed to confirm the present findings.

In summary, the present study illustrated that the hypermethylation of *PPM1G* was significantly associated with the impulsive choice of risk-taking propensity, not the severity of problematic alcohol drinking, in a male Korean clinical population with AUD. The present finding supports the conclusion that the altered methylation status of *PPM1G* influences the pathophysiology of AUD, possibly through the impulsivity trait of risk-taking. Further prospective research is required in order to determine the role of *PPM1G* in the pathophysiology of AUD and multidimensional impulsivity.

## Supplementary information

Supplementary Tables.
