# Publisher Correction: Association of *PPM1G* methylation with risk-taking in alcohol use disorder

**DOI:** 10.1038/s41598-020-67130-2

**Published:** 2020-06-18

**Authors:** Chun Il Park, Hae Won Kim, Syung Shick Hwang, Jee In Kang, Se Joo Kim

**Affiliations:** 1 Institute of Behavioral Science in Medicine, Yonsei University College of Medicine, Seoul, Republic of Korea; 2 Department of Psychiatry, Yonsei University College of Medicine, Seoul, Republic of Korea; 3 Department of Medical Education, Yonsei University College of Medicine, Seoul, Republic of Korea; 4 Graduate School, Yonsei University College of Medicine, Seoul, Republic of Korea

Correction to: *Scientific Reports* 10.1038/s41598-020-62504-y, published online 26 March 2020

In the original version of this Article, the author Chun Il Park was incorrectly indexed. This error has now been corrected.

