# Supplementary material for: Association of PPM1G methylation with risk-taking in alcohol use disorder
Source: Sci Rep. 2020 Mar 26;10:5490. doi: 10.1038/s41598-020-62504-y (PMC7099006; doi:10.1038/s41598-020-62504-y)
Supplement: Supplementary file 1 — Supplementary Tables. [file 41598_2020_62504_MOESM1_ESM.docx]

**Supplementary tables**

**Title**

Association of PPM1G methylation with risk-taking in alcohol use disorder

**Authors**

Chun Il Park^1,2^, Hae Won Kim^1,3^, Syung Shick Hwang^4^, Jee In Kang^1,2^ & Se Joo Kim^1,2^

^1^Institute of Behavioral Science in Medicine, Yonsei University College of Medicine, Seoul, Republic of Korea;

^2^Department of Psychiatry, Yonsei University College of Medicine, Seoul, Republic of Korea;

^3^Department of Medical Education, Yonsei University College of Medicine, Seoul, Republic of Korea

^4^Graduate School, Yonsei University College of Medicine, Seoul, Republic of Korea

| Table S1. Multiple linear regression analysis^a^ with enter method for predicting the AUDIT score in patients with AUD (N = 244) | | | | | |
| --- | --- | --- | --- | --- | --- |
|  | B | SE | β | T | P |
| Constant | 33.827 | 28.759 |  | 1.176 | 0.241 |
| Age | -0.147 | 0.073 | -0.13 | -2.003 | 0.046 |
| Duration of AUD | 0.164 | 0.053 | 0.203 | 3.101 | 0.002 |
| BDI | 0.143 | 0.045 | 0.235 | 3.173 | 0.002 |
| BAI | 0.083 | 0.05 | 0.122 | 1.655 | 0.099 |
| ^b^Early life trauma | 2.127 | 0.92 | 0.141 | 2.312 | 0.022 |
| ^c^*PPM1G* methylation (%) | -0.102 | 0.305 | -0.019 | -0.333 | 0.739 |
| ^a^ Model summary: R^2^ = 0.207, adjusted R^2^ = 0.187, F_(6, 237)_ = 10.286, P < 0.001 | | | |  |  |
| ^b^ High early life trauma was coded as 1, low early life trauma was coded as 0 based on the median value of mPCCTS | | | | | |
| ^c^ Mean value of methylation at three CpG sites in *PPM1G* | | |  |  |  |
| AUDIT, Alcohol Use Disorder Identification Test; AUD, alcohol use disorder; SE, Standard error; BDI, Beck Depression Inventory; BAI, Beck Anxiety Inventory | | | | | |

| Table S2. Multiple linear regression analysis^a^ with enter method for predicting the BIS score in patients with AUD (N = 244) | | | | | |
| --- | --- | --- | --- | --- | --- |
|  | B | SE | β | T | P |
| Constant | 59.527 | 38.158 |  | 1.56 | 0.12 |
| Age | -0.064 | 0.097 | -0.04 | -0.653 | 0.514 |
| Duration of AUD | 0.112 | 0.07 | 0.099 | 1.593 | 0.112 |
| BDI | 0.326 | 0.06 | 0.386 | 5.46 | <0.001 |
| BAI | 0.114 | 0.067 | 0.119 | 1.701 | 0.09 |
| ^b^Early life trauma | 2.134 | 1.221 | 0.102 | 1.747 | 0.082 |
| ^c^*PPM1G* methylation (%) | -0.193 | 0.405 | -0.026 | -0.476 | 0.635 |
| ^a^ Model summary: R^2^ = 0.276, adjusted R^2^ = 0.258, F_(6, 237)_ = 15.092, P < 0.001 | | | |  |  |
| ^b^ High early life trauma was coded as 1, low early life trauma was coded as 0 based on the median value of mPCCTS | | | | | |
| ^c^ Mean value of methylation at three CpG sites in *PPM1G* | | |  |  |  |
| BIS, Barratt Impulsiveness Scale; AUD, alcohol use disorder; SE, Standard error; BDI, Beck Depression Inventory; BAI, Beck Anxiety Inventory | | | | | |

| Table S3. Multiple linear regression analysis^a^ with enter method for predicting the stop signal reaction time (SSRT) in patients with AUD (N = 221) | | | | | |
| --- | --- | --- | --- | --- | --- |
|  | B | SE | β | T | P |
| Constant | 828.445 | 594.544 |  | 1.393 | 0.165 |
| Age | -0.311 | 1.528 | -0.015 | -0.203 | 0.839 |
| Duration of AUD | 2.81 | 1.104 | 0.194 | 2.544 | 0.012 |
| BDI | 1.128 | 0.925 | 0.103 | 1.22 | 0.224 |
| BAI | -0.725 | 1.037 | -0.059 | -0.699 | 0.485 |
| ^b^Early life trauma | -5.876 | 19.27 | -0.022 | -0.305 | 0.761 |
| ^c^*PPM1G* methylation (%) | -7.364 | 6.304 | -0.078 | -1.168 | 0.244 |
| ^a^ Model summary: R^2^ = 0.053, adjusted R^2^ = 0.027, F_(6, 214)_ = 2.001, P = 0.067 | | | |  |  |
| ^b^ High early life trauma was coded as 1, low early life trauma was coded as 0 based on the median value of mPCCTS | | | | | |
| ^c^ Mean value of methylation at three CpG sites in *PPM1G* | | |  |  |  |
| AUD, alcohol use disorder; SE, Standard error; BDI, Beck Depression Inventory; BAI, Beck Anxiety Inventory | | | | | |
